# Wearable technology for spine movement assessment: A systematic review

**DOI:** 10.1016/j.jbiomech.2017.09.037

**Published:** 2017-11-07

**Authors:** Enrica Papi, Woon Senn Koh, Alison H. McGregor

**Affiliations:** a Department of Surgery and Cancer, Imperial College London, London, UK; b Department of Bioengineering, Imperial College London, London, UK

**Keywords:** Spine, Wearable sensor, Motion analysis, Kinematics, Kinetics

## Abstract

Continuous monitoring of spine movement function could enhance our understanding of low back pain development. Wearable technologies have gained popularity as promising alternative to laboratory systems in allowing ambulatory movement analysis. This paper aims to review the state of art of current use of wearable technology to assess spine kinematics and kinetics.

Four electronic databases and reference lists of relevant articles were searched to find studies employing wearable technologies to assess the spine in adults performing dynamic movements. Two reviewers independently identified relevant papers. Customised data extraction and quality appraisal form were developed to extrapolate key details and identify risk of biases of each study. Twenty-two articles were retrieved that met the inclusion criteria: 12 were deemed of medium quality (score 33.4–66.7%), and 10 of high quality (score >66.8%). The majority of articles (19/22) reported validation type studies. Only 6 reported data collection in real-life environments. Multiple sensors type were used: electrogoniometers (3/22), strain gauges based sensors (3/22), textile piezoresistive sensor (1/22) and accelerometers often used with gyroscopes and magnetometers (15/22). Two sensors units were mainly used and placing was commonly reported on the spine lumbar and sacral regions. The sensors were often wired to data transmitter/logger resulting in cumbersome systems. Outcomes were mostly reported relative to the lumbar segment and in the sagittal plane, including angles, range of motion, angular velocity, joint moments and forces.

This review demonstrates the applicability of wearable technology to assess the spine, although this technique is still at an early stage of development.

## 1 Introduction

Low back pain (LBP) has been identified as a major cause of adult disability contributing to substantial limitations in daily, recreational and occupational activities (Hoy et al., 2014). Despite LBP being a common problem, there is still a lack of understanding of what causes it in the absence of identifiable underlying pathology, making its management controversial. This is the case for non-specific LBP which accounts for up to 85% of all cases (Dankaerts et al., 2006). However, it is believed that the cause of LBP is multi-factorial involving both mechanical and psychosocial factors (Van Dillen et al., 2007, Clays et al., 2007). Whilst the latter are measured through questionnaires and scores reflecting patients’ subjective views, there is still an open debate on mechanical factors and their contribution. To obtain a better insight into the mechanical factors, an understanding of spine movement patterns during daily tasks has been recommended (Hernandez et al., 2017, Mitchell et al., 2008, Van Dillen et al., 2007). Daily tasks may impart, when impaired, abnormal, repetitive and prolonged stresses on the spine which may lead to LBP (Hernandez et al., 2017). Continuous monitoring of spinal movement provides the opportunity for objective and quantitative assssemnt of kinematics and/or kinetics which provides an insight into how spine movements influence mechanical changes and hence LBP development and persistence. In this way the link between LBP and daily activities can be assessed. However, recent recommendations advocate to not limit the analysis to the affected spine regions but to consider the whole body kinematic chain (McGregor and Hukins, 2009, Muller et al., 2015). Since lower limb kinematic/kinetic assessment with both laboratory and portable technologies is widely documented in the literature, this study is focused on spinal movement assessment.

Spinal movement analysis techniques have evolved mainly around the use of 3D motion tracking systems and electromagnetic tracking devices in a laboratory environment (Mitchell et al., 2008, Muller et al., 2015). These systems provide complex descriptions of body segment movement but only provide snap-shots of the subject over a short period of time, have limited capture volume constrained by camera positioning or receiver-magnetic field source distance, and create artificial environments for movement assessment. Therefore, despite the detailed information obtained these systems fail to reflect real-life situations.

Conversely, in-field measurements could aid assessment, objectify treatment pathways, and mitigate risks exposure. They will also support monitoring and treatment delivery thus facilitating and encouraging patients’ self-management. LBP is often related with the workplace and therefore is best understood through in-field measurements. These will facilitate our understanding of works ergonomics and the relationship between environment and LBP.

Advancements in miniaturised technology have brought about new possibilities for long-term monitoring of body movement including assessments in real-life environments. Portable and wearable sensors, in particular inertial sensors, have been introduced and have rapidly gained popularity in biomechanical studies of movement (Bonato, 2010). Properties such as light-weight, small size, low cost, energy efficiency and portability make these sensors suitable for a variety of applications, from simply monitoring activities of daily living (Yang and Hsu, 2010) or walking speed (Yang and Li, 2012) to more complex body segment kinematics estimation, particularly for the lower limbs (Fong and Chan, 2010), gait analysis (Shull et al., 2014) and balance assessment (Hubble et al., 2015).

In the context of LBP, and in the interest of capturing spine movement in real-life settings, this review aims to understand the state of art of current use of wearable technologies, for the assessment of spine kinematics and kinetics.

## 2 Methods

### 2.1 Search strategy

A systematic search of the following electronic databases was performed from inception up until August 2016: PubMed, Embase, ACM, Scopus and IEEEXplore. The search focused on identifying articles that included terms under the following general categories: sensors, wearable, outcome, spine. The search terms used are shown in Table 1. Hand searching and screening the reference lists of relevant articles were also performed to identify articles that may have been overlooked by the electronic searches. Retrieved articles were imported into EndNote X7 software (Thomson, Reuters, Carlsbad, CA).

**Table 1:** Search terms used in the systematic review.

General	Specific search terms
Sensors	Sensor OR sensors OR sensing OR inertia OR inertial OR accelerometer OR gyroscope OR goniometer OR goniometry OR electrogoniometer OR “smart textile” OR “body sensor network” AND
Wearable	Wearable OR portable OR movable OR worn OR ambulatory OR “non-invasive” OR “body-mounted” AND
Outcome	Kinetic OR kinetics OR kinematic OR motion OR motions OR movement OR assessment OR “joint angle” AND
Spine	Spine OR spinal OR back OR cervical OR thoracic OR lumbar OR vertebra

### 2.2 Inclusion and exclusion criteria

The titles and abstracts of articles retrieved from the searches were assessed independently by two reviewers (EP, WSK). Full text of potential articles was assessed against eligibility criteria by the two reviewers independently. Inclusion and exclusion disagreements were resolved by consensus. Articles were included if they satisfied the following criteria: were published in English; assessed the spine, including the lumbar spine, using wearable technologies; were peer-reviewed; included at least one of following as outcome measures: spine kinematics, kinetics, and posture parameters as obtained from wearable technology; involved an adult population (≥18 years old) performing dynamic movement tasks. Articles were excluded if: they were review or case-study; they used only non-wearable devices; wearable technology was used to only quantify physical activity or spatio/temporal parameters of the activity performed; they described a potential technology not validated/used with human subjects; they did not assess motion of the spine.

### 2.3 Data extraction and quality appraisal

Customised data extraction and quality appraisal forms were developed to extrapolate key details and identify risk of biases of each study. The following details were extracted: study design, sample size, participants’ demographics (e.g. population type, age, gender, mass, height), tasks conducted, measuring system used, data sampling, participant set-up (e.g. positioning of the sensors, fixation method), data processing (e.g. filter use for the signal), kinematic and kinetic variables evaluated from sensors signals, statistical analysis technique, reliability/accuracy evaluation.

The quality of the selected studies was assessed using a customised checklist as no applicable standardised guidelines were identified. The checklist was however developed based on tools previously used in motion analysis reviews to include elements related to external validity (e.g. sampling methods and participants description), and biases in protocol description and outcomes reporting, with specific questions to assess information relating to technology use and signal evaluation (Dobson et al., 2007, Needham et al., 2016). The quality checklist consisted of 20 items (Table 2); each item was rated as zero (no detail), one (limited detail) and two (good detail).

**Table 2:** Quality assessment results of included articles.

Quality Index Item number	Bartalesi et al. (2010)	Baten et al. (1996)	Boocock et al. (1994)	Charry et al. (2011)	Chhikara et al. (2010)	Consmuller et al. (2012)	Dunk and Callaghan (2010)	Faber et al. (2016)	Goodvin et al. (2006)	Ha et al. (2013)	Khurelbaatar et al. (2015)	Lee and Park (2011)	Lee et al. (2011a)
1	2	2	2	2	2	2	2	2	2	2	2	2	2
2	0	0	1	0	1	0	0	0	0	2	2	2	0
3	0	0	1	0	0	2	2	2	0	2	2	2	2
4	0	0	2	1	0	2	2	0	2	2	0	1	2
5	0	0	0	0	0	1	0	0	0	0	0	0	0
6	0	0	0	0	0	0	0	0	0	0	0	0	0
7	2	1	2	2	2	2	2	2	2	2	1	2	2
8	0	0	1	0	0	0	0	0	0	2	0	0	0
9	0	0	2	1	1	2	2	2	2	1	0	2	1
10	1	1	2	2	2	2	1	1	2	2	1	2	2
11	2	2	2	2	2	2	2	1	2	2	1	2	2
12	2	2	2	1	2	2	0	1	2	2	1	2	2
13	2	1	2	1	2	2	2	1	2	2	2	2	2
14	2	1	1	2	1	1	2	1	1	1	1	2	2
15	1	2	2	2	2	1	2	1	2	2	2	2	2
16	2	2	2	2	2	0	0	2	2	2	2	0	0
17	2	2	2	2	2	1	0	2	1	2	2	1	1
18	1	1	2	2	2	2	2	2	2	2	2	2	2
18	1	0	2	2	2	2	2	2	1	2	2	2	2
20	0	0	0	0	1	2	0	2	0	2	2	1	1

Total score (/40)	20	17	30	24	26	28	23	24	25	34	25	29	27
Percentage score	50	43	75	60	65	70	58	60	63	85	63	73	68
Quality category	M	M	H	M	M	H	M	M	M	H	M	H	M

Quality Index Item number	Lee et al. (2011b)	Morlock et al. (2000)	Nevins et al. (2002)	O’Sullivan et al. (2012)	Thoumie et al. (1998)	Van Hoof et al. (2012)	Walgaard et al. (2016)	Wong and Wong (2008a)	Wong and Wong (2008b)	Paper with full score	Paper with half score	Total	Half score paper %	Full score paper %	Total %
1	2	2	2	2	2	2	2	2	2	22	0	22	0	100	100
2	0	2	0	2	1	1	1	1	1	5	7	12	32	23	55
3	2	2	0	2	2	2	2	2	2	15	1	16	5	68	73
4	2	1	0	2	1	2	1	0	0	9	5	14	23	41	64
5	0	1	0	1	0	2	0	0	0	1	3	4	14	5	18
6	0	2	0	0	0	0	0	0	0	1	0	1	0	5	5
7	2	2	2	2	1	2	2	2	2	19	3	22	14	86	100
8	0	2	0	0	1	2	0	0	0	2	2	4	9	9	18
9	1	0	2	2	0	1	2	0	0	9	6	15	27	40	68
10	2	2	1	2	2	2	1	2	2	15	7	22	32	68	100
11	2	2	1	2	2	2	2	2	2	19	3	22	14	86	100
12	2	2	2	2	2	2	2	2	2	18	3	21	14	82	96
13	2	2	0	2	1	2	1	2	2	16	5	21	23	73	96
14	2	2	1	1	1	1	2	1	1	8	14	22	64	36	100
15	2	2	1	2	2	2	2	2	2	18	4	22	18	82	100
16	1	2	0	2	2	2	2	2	2	16	1	17	5	73	77
17	1	1	1	2	2	2	2	1	2	13	8	21	36	59	95
18	2	2	1	2	2	2	2	2	2	19	3	22	14	86	100
18	2	2	0	2	2	2	2	2	2	18	2	20	9	82	91
20	1	2	0	2	0	2	2	2	2	9	4	13	18	40	59

Total score (/40)	28	35	14	34	26	35	30	27	28						
Percentage score	70	88	35	85	65	88	75	68	70						
Quality category	H	H	M	H	M	H	H	M	H						

M:Medium, H:High.

1. Were the research objectives or aims clearly stated?

2. Was the study design clearly described?

3. Was the study population adequately described?

4. Were the eligibility criteria specified?

5. Was the sampling methodology appropriately described?

6. Was the sample size used justified?

7. Did the method description enable accurate replication of the measurement procedures?

8. Was the participants’ assessor described (e.g. expertise)?

9. Was a system for standardizing movement instructions reported?

10. Was the equipment design and set up clearly described?

11. Were sensors locations accurately and clearly described?

12. Was sensor attachment method clearly described?

13. Were the spine segments analysed clearly described?

14. Was the signal/data handling described?

15. Were the main outcomes measured and the related calculations (if applicable) clearly described?

16. Was the system compared to an acknowledged gold standard?

17. Were measures of reliability/accuracy of the equipment used reported?

18. Were the main findings of the study stated?

19. Were the statistical tests appropriate?

20. Were limitations of the study clearly described?

## 3 Results

### 3.1 Articles selection

**Fig. 1:**
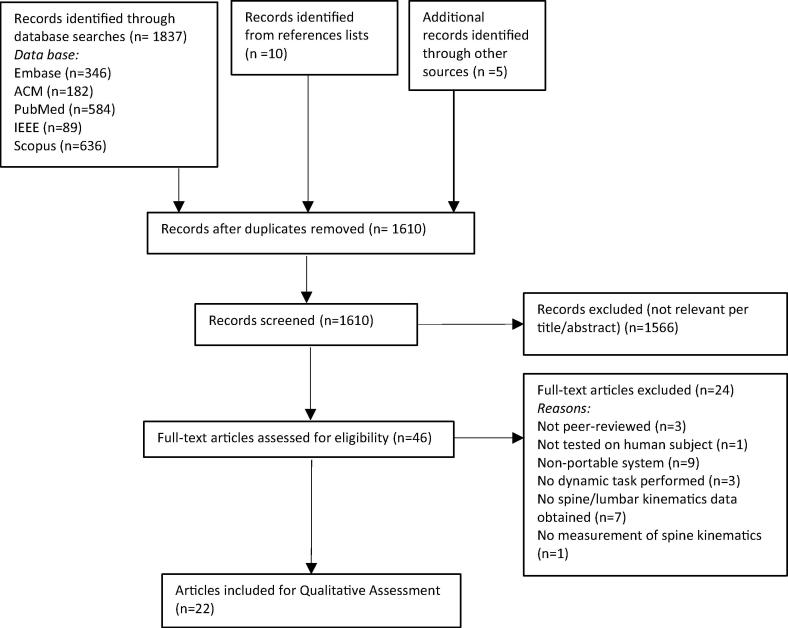
Flow diagram depicting the review process.

The search identified 1837 potentially relevant articles with 15 articles identified from references of related articles and hand searches. 1610 articles remained for consideration after removing duplicates. Following screening of title and abstract, full texts of 46 articles were retrieved. Twenty-two articles satisfied the inclusion criteria. The articles selection process and reasons for full-text articles exclusion are shown in Fig. 1.

### 3.2 Quality of reviewed articles

The overall quality of papers was rated according to Ratcliffe et al. (2014), 12 papers were deemed of medium quality (score 33.4–66.7%), and 10 of high quality (score >66.8%) (Table 2). There was a significant low rate of addressing a few core methodological questions: sample size was justified in only 1 paper; 4 papers described sampling methodology; assessor expertise was reported in 4 papers; participants’ eligibility criteria were fully described in 9 and partly in 5 papers, standardised movement instructions and limitations were reported in 14 and 13 papers respectively. Higher quality, on the other hand, was generally recorded in reporting research objectives, and in the description of the methods and systems used. Comparison with a gold standard system was described in 17 papers and measures of accuracy/reliability of the system used in 21 papers.

### 3.3 Descriptive aspects of reviewed articles

In three studies (Dunk and Callaghan, 2010, Lee et al., 2011b, Van Hoof et al., 2012), the aim was to compare spinal motion differences between healthy and LBP groups. All other studies aimed at introducing and/or validating portable assessment tools for spine movement evaluation. The sample size of the papers reviewed was small with 10 of them considering up to 10 participants (Baten et al., 1996, Bartalesi et al., 2010, Charry et al., 2011, Chhikara et al., 2010, Faber et al., 2016, Goodvin et al., 2006, Khurelbaatar et al., 2015, Lee and Park, 2011, Nevins et al., 2002, Wong and Wong, 2008a), including 4 in which only one participant was involved. In only 4 studies, the sample size was greater than 25 (Consmüller et al., 2012, Dunk and Callaghan, 2010, Ha et al., 2013, Walgaard et al., 2016).

Most of the studies assessed spine motion in healthy young adult subjects. Five studies also recruited LBP participants (Dunk and Callaghan, 2010, Lee et al., 2011a, Lee et al., 2011b, Morlock et al., 2000, Van Hoof et al., 2012).

Range of motion (ROM) manoeuvres were common tasks performed by the participants, however tasks such as lifting (2/22) (Baten et al., 1996, Charry et al., 2011), seated computer use (1/22) (Dunk and Callaghan, 2010), cycling (1/22) (Van Hoof et al., 2012), walking (2/22) (Khurelbaatar et al., 2015, Lee and Park, 2011), sit-to-stand (2/22) (Walgaard et al., 2016, Wong and Wong, 2008b) and stairs climbing (2/22) (Lee and Park, 2011, Lee et al., 2011b) were also considered. Some studies monitored participants in their working environments (3/22) (Boocock et al., 1994, Morlock et al., 2000, Thoumie et al., 1998) and during everyday activities in real-life contexts (3/22) (Nevins et al., 2002, Wong and Wong, 2008a, Wong and Wong, 2008b) and simulated in laboratories (3/22) (Goodvin et al., 2006, Nevins et al., 2002, O’Sullivan et al., 2012).

### 3.4 Sensors specification and set-up

Details of sensors described in each paper are reported in Table 3.

**Table 3:** Sensors specifications, set-up and outcomes recorded for the included articles.

Articles	Units	Type	Size and Weight	Data Transmission/Storage	Sample Frequency	Data Filtering	Sensors Placement	Spine Outcomes reported	Gold Standard
Bartalesi et al., 2010	3	Newly developed system composed by: textile piezoresistive sensor (single strip of conductive elastometer) and 2 tri-axial accelerometers (LIS3L02Al by ST Microelectronics)	–	–	–	128 th order Finite Impulse Response (FIR) filter	Accelerometers: T12, Sacrum. Piezoresistive strip over the lumbar spinous processes	Length of lumbar arch	BTS Elite4 optical motion capture system (BTS Bioengineering Corp., New York, USA)
Baten et al.,1996	1	Accelerometer (IC sensors™), miniature rate gyroscope (Murata™), sensors mounted on solid strip of orthoplast material	–	–	–	–	L5/S1	Actual back absolute angle; absolute inclination of the line between L5/S1 and the shoulder from gyroscope signal; semi-stationary estimation of the absolute inclination of the same line as above	Vicon optical motion capture system (Oxford Metrics, Oxford, UK)
Boocock et al., 1994	2	Flexible electrogoniometer (2 planes measurement) (Penny and Giles, Blackwood, UK)	48 g	Data logger: 156 g pocket sized module; 65 K memory (27.3 min continuous data storage)	20 Hz	–	One end on spinous process of S1 and S2, and second end over T12	Sagittal angle of lumbar curvature	Fluid-filled inclinometer (MIE Medical Research Ltd. Leeds, UK), and draftsman's flexicurve (Burton 1986)
Charry et al., 2011	2	Newly developed system ‘ViMove’ composed by: 3D accelerometer (Bosch SMB380) with Serial Peripheral Interface (SPI) digital output and low noise 1D gyroscope (Epson XV8100)	–	Wireless to base station (256 Mb)	20 Hz	2nd order Butterworth filter, 5 Hz cut-off frequency	L1, S1	3D lumbar angles	NDI Optotrack System (Northen Digital, Waterloo ON, Canada)
Chhikara et al., 2010	2	Newly developed system ‘IMPAIRED’ composed by: [BSN Development Kits v3] tri-axial accelerometer (Analog Devices ADXL330), tri-axial gyroscope formed by 2 perpendicularly mounted dual axis gyroscopes (Invensense IDG300A) *only one axis connected*	–	Wireless to Base station, 15 m range, 1 h battery life	25 Hz	–	L1, L5/S1	Lumbar and pelvic angular velocities, Lumbar and pelvic angles and range of motions (sagittal plane of movement)	NDI PolarisVicra optical tracking system (Northen Digital, Waterloo ON, Canada)
Consmuller et al., 2012	2	Epionics SPINE (Epionics Medical GmbH, Potsdam, Germany): Strain-gauge sensor strips with 3D accelerometer at lower end of each strip	120 g	Data logger wired to sensors	50 Hz	–	Paravertebrally 5 cm away from spinal column with caudal segment on PSIS	Lordosis/kyphosis angle, Lumbar range of motion (sagittal plane of movement)	–
Dunk and Callaghan, 2010	2	Tri-axial accelerometer (S2-10G-MF, NexGen Ergonomics, Montreal, Quebec, Canada)	–	–	128 Hz	4 th order Butterworth filter, 1 Hz cut-off frequency	L1, S2	Lumbar spine angle, Lumbar range of motion, Shift (step-like angular change in lumbar angle), Fidgets(small rapid changes of the lumbar angle about the same average position)	–
Faber et al., 2016	17	Xsens MVN full body inertia/magnetic motion capture system (Xsens technologies BV, Enschede, Netherlands)	–	–	120 Hz	–	Pelvis, Head, upper arms, forearm, thighs, shank, feet, scapulae, sternum, hand	3D L5/S1 moment time series and peaks; ground reaction forces	Certus Optotrack optical motion capture system (Northen Digital, Waterloo ON, Canada) 2 Kistler force platforms (Kistler Instrumente AG, Winterthur, Switzerland)
Goodvin et al., 2006	3	MT9 sensors (Xsens technologies BV, Enschede, Netherlands)	–	XBus device wired to sensors, data transferred via serial cable to PC from XBus	–	–	Head, C7/T1, L4-5	Cervical, thoracic and lumbar flexion/extension and lateral bending angles, roll, pitch and yaw motions	Vicon optical motion capture system (Oxford Metrics, Oxford, UK)
Ha et al., 2013	2	MTx sensors (Xsens technologies BV, Enschede, Netherlands)	38 × 53 × 21 mm; 30 g	XBus wired to sensors, Bluetooth data transfer to a PC from XBus	100 Hz		L1, S1	Lumbar 3D range of motion	ElectromagneticFastrak system (Polhemus, USA)
Khurelbaatar et al., 2015	17	Xsens MVN full body inertia/magnetic motion capture system (Xsens technologies BV, Enschede, Netherlands) Pedar-X in-shoe pressure measurement system (Novel GmbH, Munich, Germany)	–	–	–	–	Head, trunk, pelvis, upper and lower extremities	Cervical, thoracic, lumbar joint forces, joint moments	Hawk® motion capture system (Motion Analysis, Santa Rosa, CA, USA) MP4060® force platform (Bertec Corporation, Columbus, OH, USA)
Lee and Park, 2011	5	MTx sensors (Xsens technologies BV, Enschede, Netherlands)	–	XBus wired to sensors, Bluetooth data transfer to a PC from XBus	100 Hz	Xsens Kalman filter algorithm	T1, T12, S1, and 2 sensors 6 cm above each ankle in alignment with the fibula	Thoracic, Lumbar, Pelvis 3D angles, range of motions	–
Lee et al., 2011a	3	MTx sensors (Xsens technologies BV, Enschede, Netherlands)	–	XBus wired to sensors, Bluetooth data transfer to a PC from XBus	50 Hz	Xsens Kalman filter algorithm	T1, T12, S1	Thoracic, Lumbar, Pelvis flexion/extension angles, range of motions, angular velocities	–
Lee et al., 2011b	5	3 MTx sensors and 2 MT9 (Xsens technologies BV, Enschede, Netherlands)	–	XBus wired to sensors, Bluetooth data transfer to a PC from XBus	50 Hz 100 Hz	Xsens Kalman filter algorithm	T1, T12, S1 and two over Shank above each ankle	Thoracic, Lumbar, Pelvis 3D angles, range of motion	–
Morlock et al., 2000	8	1 Uni and 7 bi-axial goniometers (Penny and Giles, Gwent, UK) Pedar in-shoe pressure measurement system (Novel GmbH, Munich, Germany)	–	24-channel data logger (BIOSTORE, Wehrheim, Germany) connected via cables to the goniometers, RAM card with 45 min max duration. Pedar data stored onto the hard-disk of a laptop connected to the subject via a 10 m cable.	10 Hz 20 Hz (Pedar system)	4 th order Butterworth filter, 1.8 Hz cut-off frequency	Inferior of S1, Superior of T12, Lower limbs	3D lumbar spine angles, 3D joint moment at L5/S1, Bone to Bone contact force at L5/S1	Vicon optical motion capture system (Oxford Metrics, Oxford, UK)
O’Sullivan et al., 2012	1	BodyGuard™ spinal posture monitoring device (Sels Instruments, Belgium) (Strain gauge unit)	–	Battery powered processing unit, wireless communication to PC	20 Hz	–	Across L3 and S2	Lower lumbar extension/flexion as % strain gauge elongation, posture as % range of motion: mean and peak values	Cartesian Optoelectronic Dynamic Anthropometer (CODA™) mpx64 (Charnwood Dynamics Ltd, Leicestershire UK)
Thoumie et al., 1998	2	2D Electrogoniometer (M180, standard version, Penny and Giles, Gwent, UK)	2 × 5 cm 2 × 14 cm (End plates measures)	Digital recorder (Angle display unit, Penny and Gilles, Gwent, UK) orPortable data logger (DL 1001, Penny and Gilles, Gwent, UK) with max 163 min of recording at 3 Hz	3 Hz	–	One end plate over S1 and the second plate 10 cm apart over the lumbar spine	Lumbar flexion/extension angle maximum value and range of motion, Mean lumbar lordosis curvature	X-ray
Van Hoof et al., 2012	2	BodyGuard™ spinal posture monitoring device (Sels Instruments, Belgium) (Strain gauge unit)	10 × 28 mm measure of end pieces of strain gauge unit	Signal processing unit (56 × 71 × 15 mm). Data storage max 24 h	20 Hz	–	L3, S2	Lower lumbar flexion/extension angle as % of total lumbo-pelvic range of motion	Cartesian Optoelectronic Dynamic Anthropometer (CODA™) (Charnwood Dynamics Ltd, Leicestershire UK)
Walgaard et al., 2016	1	Single inertia sensor comprised of 3 accelerometers and 3 gyroscopes (DynaPort® Hybrid, McRoberts)	–	–	100 Hz	2nd order Butterworth filter, 15 Hz cut-off frequency	L4	Lumbar flexion range of motion, maximum flexion velocity, maximum forward velocity, forward velocity during seat-off and heel strike, maximum vertical velocity, vertical velocity at heel strike, 3D accelerations, velocities, displacements and angles	Certus Optotrack optical motion capture system (Northen Digital, Waterloo ON, Canada)
Wong and Wong, 2008a	3	Newly developed system, each sensor module composed by: Tri-axial acceleromter (KXM52-Tri-axis, Kionix) and 3 uni-axial gyroscopes (Epson)	Sensor module: 22 × 20 × 12 mm each 6 g Battery holder: 50 × 55 × 12 mm, 82 g, 8 h recording	Digital data acquisition and feedback (buzzer) system (21 × 50 × 84 mm, 44.5 g)	–	–	T1/2, T12, S1 (Elastic garment)	Average angles of thoracic and lumbar curves in the sagittal and coronal planes	Vicon optical motion capture system (Oxford Metrics, Oxford, UK)
Wong and Wong, 2008b	3	Newly developed system:each sensor module composed by: Tri-axial acceleromter (KXM52-Tri-axis, Kionix) and 3 uni-axial gyroscopes (Epson)	Sensor module: 22 × 20 × 12 mm each 6 g Battery holder: 50 × 55 × 12 mm, 82 g, 8 h recording)	Digital data acquisition and feedback (buzzer) system (21 × 50 × 84 mm, 44.5 g)	–	5th order Butterworth filter, 4 Hz cut-off frequency	T1/2, T12, S1 (Elastic straps)	Thoracic and lumbar angular velocities, thoracic and lumbar peak angles in the sagittal and coronal planes	Vicon optical motion capture system (Oxford Metrics, Oxford, UK)
Nevins et al., 2002	6	Newly developed system: sensors: Analog Devices model ADXL202E comprising 2 accelerometers in perpendicular axes and 6 satellite processors (AT90S2313)	Sensors and harness 125 g	Data logger, 32 MB compact flash card, 69 × 115 × 38 mm; 250 g	15 s epochs	–	Positioned along the vertical axis of the spine, exact location not specified	Angles relative to the vertical in each sensor in the sagittal plane	–

Newly developed and off-the shelf wearable sensors were used within the reviewed studies.

The type of sensors used to assess spine movement varied from electrogoniometers (3/22), strain gauges based sensors (3/22), textile piezoresistive sensor (1/22) and uniaxial to triaxial accelerometers, the latter often used in conjunction with gyroscopes and magnetometers (15/22). Two studies also used portable instrumented insoles in addition to the above motion sensors.

The number of sensor units used differed across the papers, with on average two sensor units positioned on the back of the participants. Positioning along the spine varied with the majority of the included studies reporting sensors placement over lumbar and sacral landmarks (L1, L3, L4, L5, S1, S2) (18/22) and T12 (8/22). Nine studies also reported positioning of the sensors on different regions of the back or body parts.

Different fixation methods were adopted, including single and double-sided adhesive tape, velcro strap, elastic belt, and neoprene strap. Some sensors were also attached using a rigid support or embedded into clothing. The size and weight of the systems used was reported in only few studies. Authors reported sampling frequencies between 3 Hz and 100 Hz, however not all authors specified the frequency used.

### 3.5 Data storage and processing

Data were collected and stored for subsequent processing in the majority of studies. Only one study described the use of real-time feedback (Goodvin et al., 2006). Data from the sensors were wirelessly transmitted to PCs/base station for data storage by the sensor units directly or, by data loggers wired to the sensors, which communicate via Bluetooth to the storage station. In eight studies, data were saved in laptops/portable data loggers attached on the participants’ bodies and wired to the sensors units (Boocock et al., 1994, Consmüller et al., 2012, Morlock et al., 2000, Nevins et al., 2002, Thoumie et al., 1998, Van Hoof et al., 2012, Wong and Wong, 2008a, Wong and Wong, 2008b). The capacity of the memory cards of the portable data loggers was seldom described.

Most of the systems required calibration before use and filtering due to noise in the data collected. This adds time to the data processing procedure before calculation of the outputs. Butterworth filters of the 2nd to the 5 th orders were adopted with cut-off frequencies ranging from 1 Hz to 30 Hz (6/22, Table 3). Kalman filters were also used (3/22) and one study used a 128 th order finite impulse response filter (Table 3). The rest of the studies did not report the type of filter used.

The sensors signals were processed to calculate different kinematic and kinetic aspects of spine movements in 3D. More commonly, however, only sagittal plane movement data were reported. Outcomes were mostly reported relative to the lumbar segment, although cervical, thoracic spine segments, and pelvis were considered in some studies. The following outcomes were reported: 3D angles, flexion/extension ROM, spine curvature sagittal angles, angular velocity, joint moments, and joint forces. Details of outcome measures for each study are shown in Table 3.

### 3.6 Reliability and accuracy

The accuracy and reliability of the systems proposed in the reviewed papers were evaluated by comparisons with gold standard systems. Optoelectronic motion capture systems were mostly used as gold standards (15/22), one study used an inclinometer (Boocock et al., 1994), and one used X-Ray (Thoumie et al., 1998). Direct comparison with a gold standard was not performed in six studies (Consmüller et al., 2012, Dunk and Callaghan, 2010, Lee and Park, 2011, Lee et al., 2011a, Lee et al., 2011b, Nevins et al., 2002). Comparisons among studies in terms of reliability and accuracy of systems used are difficult to assess as different measures were reported, they were calculated on different outcome measures and during different test circumstances. Measures of reliability and accuracy reported are shown in Table 4 for each paper.

**Table 4:** Accuracy and reliability of systems described in the reviewed articles.

Articles	Accuracy/Reliability
Bartalesi et al., 2010	2% error in length estimation; high correlation greater than 0.8 when comparing lumbar arch from reference system and new system
Baten et al.,1996	Calibration error <1%; Over 8 h accelerometer offset drift ±5%, inclination depending error 3–20% in the semi-stationary estimation of the absolute inclination; error in absolute inclination from gyroscope ±10%
Boocock et al., 1994	Calibration rig test results: 0.96 between systems angles; RMSE of 2.5° equivalent to 6° limit of agreement; Crosstalk error between 7% and 10% of the measured angle. Electrogoniometer 5.7° RMS difference, 0.78 correlation, 1.17° mean difference, intrasubject SD 4.05° between two different test occasions; Recording angle error <3 compared to gold standards; Electrogoniometer vs Fluid-filled inclinometer: RMS difference 3.89°, correlation 0.9, mean difference 1°; Electrogoniometer vs draftsman's flexicurve: RMS difference 5.87°, correlation 0.77, mean difference −1.17°
Charry et al., 2011	The RMSE achieved for one dimensional movements in the Flexion, Lateral Flexion and Twist planes were 1.0°, 0.5° and 2.4° respectively, 2.0°, 3.1°, and 5.1° for 2D movements and 2.1°, 2.4° and 4.6° for 3D movements. RMS errors averaged over the 53 movements performed by two test subjects: 1.9° and 2.1° for Flexion 2.4° and 2.1° for Lateral Flexion and 5.2° and 4.1° for Twist for Subject 1 and 2 respectively
Chhikara et al., 2010	Mean average error for angular velocity: (Lumbar) 1.52 ± 31.24, (Pelvis) 0.78 ± 9.4. Mean average error for angles: (Lumbar) −1.83 ± 1.85, (Pelvis) 0.91 ± 0.28; error in orientation results between system between 3 and 7°
Consmuller et al., 2012	Good to excellent correlation between the left and right sensors for all angles in the sagittal plane with average Pearson correlation coefficient r = 0.81. The correlation for upright standing was r = 0.85, r = 0.70 for maximum flexion, and r = 0.87 for maximum extension. The correlation for the repeated measurements on three different days was very good for segmental results during upright standing (ICC = 0.87), flexion (ICC = 0.86) and extension (ICC = 0.84), with similar results for lordosis (ICC = 0.85), flexion (ICC = 0.83) and extension (ICC = 0.79) angles. The average correlation coefficient was 0.84
Dunk and Callaghan, 2010	No accuracy/reliability measures reported
Faber et al., 2016	RMS errors were below 10 Nm for flexion, lateral flexion and twist L5/S1 moments time series. R^^2^ values > 0.993 for flexion L5/S1 moment and below 0.993 for lateral flexion and twist L5/S1 moments. ICC of the absolute peak moments were 0.971, 0.781 and 0.69 for flexion, lateral flexion and twist respectively. 3D GRF RMS errors remained below 20 N, R^^2^ above 0.981 for vertical GRF, around 0.6 for anterio/posterior and medio/lateral GRF. ICC of the vertical GRF peaks between systems was 0.998, and 0.948 and 0.559 for anterio/posterior and medio/lateral GRF peaks. During fast trunk bending overestimation of about 15 N in vertical GRF peak
Goodvin et al., 2006	From head sensor: roll, pitch and yaw average deviation 0.1°, 0.42°, and 0.2° respectively. From torso sensor: roll, pitch and yaw average deviation 0.03°, 0.06° and 0.23° respectively. For hip sensor: roll, pitch and yaw average deviation 3.1° roll, 0.33° and 1.35° respectively
Ha et al., 2013	Overall ROM R^^2^ 0.999 between systems; flexion R^^2^ = 0.7878 and correlation coefficient = 0.8876; extension R^^2^ = 0.4321 and correlation coefficient = 0.6573; right lateral flexion R^^2^ = 0.7285 and correlation coefficient = 0.8535; left lateral flexion R^^2^ = 0.8101 and correlation coefficient = 0.900; right axial rotation R^^2^ = 0.4199 and correlation coefficient = 0.6657; left axial rotation R^^2^ = 0.2633 and correlation coefficient = 0.5411. Mean differences between −0.81 and −1.26°. No significant difference between systems *p*-values greater than 0.05
Khurelbaatar et al., 2015	Bench test: single axis rotation: average RMSE 0.9 ± 0.7°; 3-axis rotation: average RMSEs were 0.8 ± 0.68° in the X-axis, 1.1 ± 0.58° in the Y-axis, and 0.8 ± 0.58° in the Z-axis. GRF normalised RMSE < 9.1% and Pearson's correlation r ≥ 0.96; Lumbar joint force normalised RMSE 5.8% and r = 0.81; Cervical joint force normalised RMSE 6.0%, r = 0.81; Thoracic joint force normalised RMSE 6.0% and r = 0.80. Moments Lumbar joint: normalised RMSE 16.9% and r = 0.86; Cervical joint: normalised RMSE 13.6% and r = 0.8; Thoracic joint normalised RMSE 8.4% and r = 0.93
Lee and Park, 2011	Not measured directly in current study. 2° RMS in dynamic motion from sensors manufacturer; Orientation error <3.1° from previous study from the same authors using MT9 sensors (which are an older version of the MTx used in this study) against a Vicon optical motion capture system
Lee et al., 2011a	Not measured directly in current study. 2° RMS in dynamic motion from sensors manufacturer
Lee et al., 2011b	Not measured directly in current study. 2° RMS in dynamic motion from sensors manufacturer. Orientation error 3.1° in roll, 0.3° in pitch, and 1.4° in yaw for MT9 sensors as measured in previous authors' study against Vicon optical motion capture system
Morlock et al., 2000	Proposed system overestimates max value of compressive Bone-to-bone (B-t-B) force by 9 ± 4%; the medio-lateral BtB force by 3 ± 58%; the antero-posterior BtB 23 ± 4%; flexion/extension moment by 12 ± 4%; the lateral flexion moment by 36 ± 7%, and the torsional moment by 67 ± 14%. Lumbar spine angles flexion/extension 1.8 ± 1% difference between systems; for torsion and lateral flexion plane the proposed system overestimated angles by more than 100%
O’Sullivan et al., 2012	Strong positive correlations and small differences between systems in sitting (Spearmans rank correlation coefficient r_s_ = 0.88 and R^^2^ = 0.78; difference 2.39°) and standing (r_s_ = 0.88 and R^^2^ = 0.78; difference 3.06°). Overall mean difference in standing and sitting <10%ROM. Agreement varied among a range of tasks in sitting and standing with differences up to a maximum of 6.2° in sit-to-stand flexion and 5.8° when putting shoe on
Nevins et al., 2002	Precision ±0.39° during static condition
Thoumie et al., 1998	Electrogoniometers repeatability ±2° between +90 and −90°; Cross talk between 2 electrogoniometers never exceeded 1° for lateral bending of less than 30°. Electrogoniometric and radiographic lumbar curve angles correlation in flexion, extension and standing positions were 0.76, 0.77 and 0.58 respectively but, the exact values were significantly different. Lumbar ROM correlation in flexion, extension and standing positions were 0.65, 0.74 and 0.48 respectively with values significantly different
Van Hoof et al., 2012	Intra- and inter-rater reliability: ICC values: 0.837–0.874 and 0.914–0.940 respectively based on previous referenced study. r_s_ = 0.88 and R^^2^ = 0.78, mean difference < 10% Flexion ROM according to another study. Additionally, the correlation between the two systems during ergometer cycling was evaluated in advance and was strong (r = 0.8), with a mean difference of 3°
Walgaard et al., 2016	RMSE < 10% for 3D lumbar accelerations, velocities, displacements and angles between systems except for sideway displacement, and non-sagittal plane rotation with RMSE 40.1 ± 47.4%. ICC ≥ 0.867 for lumbar flexion range of motion, maximum flexion velocity, maximum forward velocity, forward velocity during seat-off and heel strike, maximum vertical velocity, except for vertical velocity at heel strike ICC = 0.649. Mean absolute differences were 0.45 ± 0.35° for flexion range, 16.9 ± 16.6° for maximum flexion velocity and 0.1 ± 0.06 m/s or lower for other velocity measures
Wong and Wong, 2008a, Wong and Wong, 2008b	(Bench test) Static measurement: RMS difference <1°; Pearson’s correlation coefficient for angles >0.999. Dynamic tilting measurement: RMS difference <1.5°; Pearson’s correlation coefficient for angles >0.999, RMS angular velocity along the x-axis 35.2 ± 1.9°/s and along y-axis 34.1 ± 1.7°/s
Wong and Wong, 2008a, Wong and Wong, 2008b	(Bench test) Static measurement: RMS difference <1°; Pearson’s correlation coefficient for angles >0.999. Dynamic tilting measurement: RMS difference <1.5°; Pearson’s correlation coefficient for angles >0.999, RMS angular velocity <40°/s. (Participants tests) RMS differences between systems were <3.1° for the sagittal plane and <2.1° for the coronal plane at both thoracic and lumbar regions and movements (flexion, left and right lateral bending, stand-sit-stand). The correlation coefficients of the measurements were all above 0.829

## 4 Discussion

Use of wearable sensor based motion capture systems for the quantitative assessment of human movement is rapidly growing. Portable systems present as a practical choice for in-field measurements, thereby enabling analysis in real-life settings where movement disorders may develop and perpetuate. This is the case with LBP, notably one of the most frequent claimed occupational disorders (Kerr et al., 2001, Dagenais et al., 2008). In-field assessment, therefore, may positively impact on the healthcare provision of LBP through enhanced understanding of the underlying mechanics.

This systematic review aimed to examine the literature to understand the state of art in relation to the use of wearable technology to assess spine kinematics and kinetics, with the view to use them for clinical trials and work-based assessments. Twenty-two articles were found to satisfy the inclusion criteria. The appraisal of studies quality revealed that the overall scientific quality of the reviewed papers is of medium to high quality. However, it was found that papers were lacking a thorough description of their sample in terms of calculation and justification of the sample size, participants’ inclusion criteria, and assessors’ expertise. The use of standardised instructions for the experiments was unclear. On the other hand, studies performed well in elements related to the description of the technology used, experimental protocol and outcomes.

The small number of existing literature found on portable assessment of spine motion revealed limited adoption of this technique, which is at an early stage of development and translation. The majority of papers, reported validation type studies with a proof-of-concept theme. New and off-the-shelf technologies were described and validated against more traditional motion capture systems to assess spine movement, as a first step to demonstrate the potential of such technologies for clinical and daily use. Validation mainly occurred with small groups of healthy young participants; only 5 studies explored wearable sensors use with clinical populations. Moreover, the majority of studies were conducted in research laboratories, although some papers described data assessment in working environments and daily living. Boocock et al. (1994) used a flexible electrogoniometer to track changes in the lumbar spinal sagittal curvature in 4 garage mechanics over 2 h of their working day. An electrogoniometer was also used by Thoumie et al. (1998) to assess changes in lumbar flexion/extension when nurses and physiotherapists wore a belt during daily shifts. In Morlock et al. (2000) a full body portable system comprising of 8 electrogoniometers and pressure distribution insoles was used to determine lumbar spine loads and 3D lumbar angles in nurses, with and without LBP, during 4 h of continuous monitoring. A smart garment integrating a tri-axial accelerometer and 3 uni-axial gyroscopes for posture training was developed and used in two studies (Wong and Wong, 2008a, Wong and Wong, 2008b) with 5 healthy participants over 4 and 3-day trials during everyday activities. Sagittal and coronal plane angles of the thoracic and lumbar spine were estimated in these studies. Finally, Nevins et al. (2002) monitored spinal posture in one subject while day-to-day activities took place. These exceptions demonstrate, although still at a preliminary stage, the possibility to use these systems for in-field measurements.

When studies were performed in research laboratories, participants performed mostly ROM tasks even though more dynamic activities (e.g. walking) were assessed. The validity of using such technologies in dynamic everyday tasks is important for translation into real-life use. Such technologies in fact may reveal impractical during dynamic tasks in daily living in terms of positioning and sustainability of sensor attachment that will be critical for the robustness of the outputs. Moreover, the presence of wires may cause discomfort for long-term use and restrict natural movements, jeopardising data acquisition. Wires were used in the systems described to connect the sensor units to data loggers or directly to PCs compromising their wearability and practical translation. Alternatively, Bluetooth connection was used to transfer data to a stationary PC. In those cases, however, the systems cannot be considered entirely portable as a recording station is still required and the subject will always need to be within a certain distance to maintain Bluetooth connectivity. This could be obviated if a data logger is used. Data loggers, hence, represent comprises for portability despite adding substantial volume to the measurement system worn. Data logging is one area to be improved in the development of such technology in terms of miniaturisation, Bluetooth connectivity with the sensor units and memory capacity.

In the majority of studies, the systems used consisted of inertia-based sensors in the form of accelerometers, single or three axes, used alone or together with gyroscopes or gyroscopes and magnetometers in off-the-shelf inertia measurement units. Accelerometers and gyroscopes are known to be affected by drift errors when acceleration and angular velocity signals are integrated to obtain positions and orientations values, whilst magnetometers signals can be distorted by surrounding magnetic fields (Alonge et al., 2014). Other sources of errors are related to sensors positioning: skin movement artefact and misalignment between the sensor axes and underlying anatomical segments. The other types of systems described, electrogoniometer and strain gauges based sensors, are also sensitive to the correct sensor positioning, yet not affected by the presence of magnetic fields and post-processing analysis as they provide direct measures of angles. Despite this, technology developments have favoured the uptake of inertia sensors due to their small size and versatility. Moreover, spine movement is not isolated to one anatomical plane. Inertia sensors facilitate the characterisation of LBP related movement disorders by allowing 3D analysis. Despite the potential errors outlined, all systems showed good to excellent agreement and correlation with gold standards for the outcomes evaluated. Gold standards were represented, in the majority of the included studies, by optoelectronic motion capture systems which are frequently used to assess human movement. Measures of sensor accuracy and reliability reported included the root mean square error, mean difference, intraclass correlation coefficient, Pearson correlation coefficient, Spearman’s rank correlation coefficient and coefficient of determination. From the detailed values reported in Table 4, it can be recognised that all the systems could be used to measure kinematics and kinetics parameters of the spine with good accuracy and reliability. It should be mentioned that some of these measures were reported to be related to simple tasks or bench tests. It has, however, been shown that errors can be task dependent (O’Sullivan et al., 2012) therefore it is important to evaluate the accuracy in dynamic and extreme end of range movements prior to widespread implementation. Significant errors could arise from sensor malpositioning, poor fixation, and incorrect identification of anatomical landmarks. It is important to note that such errors also impact on optical tracking systems (Leardini et al., 2005). Currently, there are no robust tools to correct for movement artefacts. As such these issues remain a limitation of non-invasive motion capture systems whether portable and not.

In terms of outcomes, all studies reported kinematics outputs, angles and/or angular velocities, but only 3 studies reported kinetics measures, joint forces and moments. The most commonly used output was the lumbar spine angle, reported as lumbar angle over time, mean and/or maximum and minimum values or in the form of ROM. Outputs were mostly described in the sagittal plane of movement and on a few occasions included measures of the cervical and thoracic spinal segments (Table 3). All outputs were obtained from data processing, including filtering, calibration, integration, mathematical calculations, that took place after the experiments thus precluding real-time biofeedback. Complex data handling algorithms mean long processing time, necessitating specialised hardware and expertise for data interpretation. This is another area that will need further work if data are to be used for clinical assessment and self-management requiring real-time feedback and fast data visualisation of clinically meaningful outputs. Real-time graphical representation of spine motion was only used by Goodvin et al. (2006). Their interface, however, need improvements to be more user friendly and clinically focused.

Overall, all the systems described in the reviewed papers are suitable to study spine movement, however the choice of the system will be dictated by the scope of the study to be conducted. Systems that require PCs and are characterised by the presence of wires are not the best candidate for every-day and prolonged use. In those cases the use of portable data storages will be advocated as described in 8 studies (Boocock et al., 1994, Consmüller et al., 2012, Morlock et al., 2000, Nevins et al., 2002, Thoumie et al., 1998, Van Hoof et al., 2012, Wong and Wong, 2008a, Wong and Wong, 2008b). However, miniaturisation and weight of the portable data logger still need to be optimised. Moreover, if spinal loading is to be measured in the field, in addition to sensors on the spine, sensors on the lower limbs will be necessary for the construction of an inverse-dynamic model as well as portable insoles for ground reaction force measurements as performed by Morlock et al., 2000, Khurelbaatar et al., 2015. If the aim of the study is to provide feedback as a mean to deliver movement intervention and retraining, a visualisation tool similar to the one described by Goodvin et al. (2006) will be needed. The systems described, as mentioned above, where mainly used for validation purposes and their capability in terms of diagnostic in LBP and other musculoskeletal conditions needs to be further explored. Although only used to quantify motion patterns differences, some studies support the use of wearable systems with LBP groups (Dunk and Callaghan, 2010, Lee et al., 2011a, Lee et al., 2011b, Morlock et al., 2000, Van Hoof et al., 2012). Worth mentioning, in this regard, it is that in clinical population, such as LBP, assessment of the spine alongside other body segments may help the diagnostic process as compensation in pain free segments may occur, so the use of a whole body portable sensor system may be required (McGregor and Hukins, 2009, Muller et al., 2015).

There are limitations to be considered when interpreting the findings of this review. Only articles published in English were included posing a language bias to article selection; the quality appraisal was performed based on the checklist developed for the study aim, a standardised tool was not found as study quality was not reported in similar reviews. Finally, the review findings are limited to the articles identified by the set search strategy.

In conclusion, this review shows the use of wearable systems to quantify spine kinematics and kinetics to date. Wearable systems represent valid tools to track spine movement, however, their use is still confined to research studies and at a preliminary stage of development and use. Data logging and processing, systems design and fixation are areas to be improved to fully exploit the wide applicability of wearable technologies overcoming grounded motion capture systems in terms of long-term monitoring and real-life assessment.

## Conflict of interest statement

Nothing to declare.
